# Progress and Challenges in Survivorship After Acute Myeloid Leukemia in Adults

**DOI:** 10.1007/s11899-022-00680-6

**Published:** 2022-09-19

**Authors:** Ginna Granroth, Nandita Khera, Cecilia Arana Yi

**Affiliations:** Mayo Clinic Arizona, Phoenix, AZ USA

**Keywords:** HRQoL, PRO, MRD, Financial burden, Allo-HCT, Survivorship care plan, Survivorship clinic

## Abstract

**Purpose of Review:**

Acute myeloid leukemia (AML) survivors face unique challenges affecting long-term outcomes and quality of life. There is scant literature on the long-term impact of AML treatment in physical and mental health, disease recurrence, and financial burden in survivors.

**Recent Findings:**

Fatigue, mental health concerns, infections, sexual dysfunction, and increase cancer recurrence occur after AML treatment. Chronic graft-versus-host disease (GVHD) and infections are common concerns in AML after hematopoietic stem cell transplantation (HCT). Survivorship guidelines encompass symptoms and complications but fail to provide an individualized care plan for AML survivors. Studies in patient-reported outcomes (PROs) and health-related quality of life (HRQoL) are sparse.

**Summary:**

Here we discuss the most common aspects pertaining to AML survivorship, late complications, care delivery, prevention of disease recurrence, and potential areas for implementation.

## Introduction

Acute myeloid leukemia (AML) is an aggressive hematologic malignancy characterized by immature myeloid cell proliferation, accumulation, and infiltration of the bone marrow, blood, and other tissues. It is one of the most common types of adult leukemia with 20,500 estimated new cases in 2022 in the United States (US) [1]. The incidence of AML is 4.2 per 100,000 per year [2] with the median age at time of diagnosis being between 65 and 70 years old [3]. The advent of new AML therapies and allogeneic hematopoietic stem cell transplant (allo-HCT) are leading to a growing population of survivors [4••]. Approximately 60–70% of adult patients aged 18–65 will achieve a complete remission, and 30.5% of these adult AML patients have a 5 year or more overall survival [1]. From 2009 to 2019, there were an estimated 28,003 AML individuals in the US who have survived 5 or more years following allo-HCT (CIBMTR, 2022). As the population of AML survivors is growing, understanding the most relevant survivorship aspects constitute a key component for routine follow-up care.

Clinicians are responsible for surveillance testing for recurrent or subsequent primary cancers, disease prevention, management of comorbid conditions, and monitoring for physical and psychological effects of cancer treatment. Although there is a significant need for information regarding AML long-term survival, quality of life, and comorbid conditions, there are limited studies and literature available to help aid with the navigation of survivorship.

We summarize the current state of AML survivorship, care delivery for AML survivors, caregiver and social support, and opportunities for improvement in research, practice, and education in AML.

### Late Effects

#### Fatigue

**Fig. 1:**
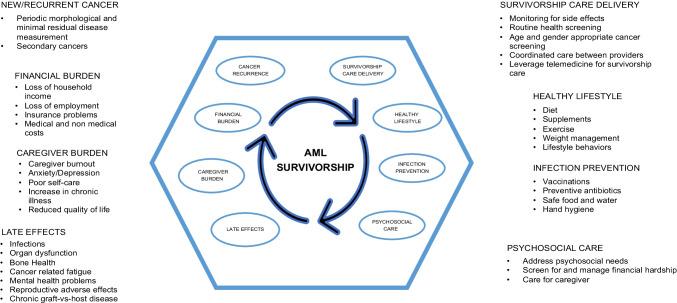
Key components in acute myeloid leukemia survivorship. This figure encompasses the key components pertinent in acute myeloid leukemia survivorship

Fatigue is a common symptom in AML survivors and is a strong predictor of worse physical, emotional functioning, and decreased survival [5]. In the AML 10 trial, 79% of patients reported fatigue after 1 year of complete remission [6]. Another study of AML survivors in remission after intensive chemotherapy demonstrated improvements in fatigue and physical function overtime in both younger and older patients [7•]. In non-transplant candidates, maintenance oral azacitidine was associated with improvement in fatigue and HRQOL [8•]. Forty-nine percent of HSCT patients in remission had difficulty taking long walks and difficulty completing household chores due to fatigue [9]. The approach to fatigue includes non-pharmacological interventions such as physical activity, monitoring of symptoms, treatment of contributing factors, and psychosocial interventions [10]. A Cochrane database review of hematologic malignancies demonstrated the benefits of exercise in HRQOL and improvement of fatigue and depression. Pharmacological options include stimulants, antidepressants, and erythropoietin. The efficacy of these agents is limited and only considered after non-pharmacological options have failed [11, 12]. Figure 1 encompasses the late effects of AML survivors, addresses psychosocial needs, and survivorship care delivery.

#### Infertility and Sexual Dysfunction

Infertility and sexual dysfunction are prevalent long-term effects in AML survivors, and clinical counseling and education should be provided prior to receiving treatment. In a self-reported fertility study of the AML survivors who wanted children after treatment, 46% of women and 40% of men felt that they had not been fully informed about fertility-related issues [13]. Many female leukemia survivors become infertile after allo-HCT [14] and have the lowest post treatment pregnancy [15]. In a meta-analysis of pregnancy rate in women after allo-HCT, it was found that 22% of women were able to become pregnant [16]. Post-allo-HCT effects in male include genital GVHD, hypogonadism, sexual dysfunction, infertility, and malignancies [17].

A cross-sectional study of sexual health and infertility, post-allo-HCT patients reported a decrease interest in sex, sexual activity, pleasure from sex, and ability to have sex [18]. Another cross-sectional study revealed that both men and women experienced loss of libido and less enjoyment of sexual activity after HSCT. This study also revealed that 72% of women experienced pain with intercourse, and 22% of these women experienced significant vulvovaginal chronic GVHD [14]. Although sexual dysfunction is a known long-term sequela of HSCT survivors, a study of 25 leading cancer centers found that 72–87% of centers had no sexual aids available for men and women to help aid in sexual dysfunction following treatment [19].

The NCCN and ASCO have established recommendations for fertility preservation in cancer patients. Sperm, oocyte, embryo, and ovarian tissue preservation are strategies supported by survivorship guidelines [20, 21]. Relevant aspects that need more study in acute myeloid leukemia include ideal timing for embryo preservation, regaining fertility after allo-HCT, health plan coverage for sexual dysfunction, and the impact of sexual health programs in leukemia survivors.

#### Infections

Acute myeloid leukemia survivors are predisposed to bacterial and fungal infections due to the duration of prolonged neutropenia, which can often lead to long-term antibiotic exposure, multi-drug resistant organisms, and the alteration of the microbiome [22]. Organisms such as *Streptococcus pneumoniae*, *Neisseria meningitidis*, *Hemophilus influenzae*, *Aspergillus*, *Candida*, *Pneumocystis jiroveci*, cytomegalovirus, and varicella zoster virus are common causes of late infections in AML survivors [23]. Due to high mortality in AML survivors, preventative therapies such as broad-spectrum antibiotics, antivirals, antifungals, and intravenous immunoglobulins (IVIG) should be utilized, especially in the setting of neutropenia or immunosuppression.

With the emergence of the COVID-19 global pandemic, AML survivors are at a higher risk of morbidity and mortality due to their overall immunocompromised state [24]. AML survivors are encouraged to get the three-dose Pfizer or Moderna mRNA COVID-19 vaccination series, as well as a 4th booster. A 5th booster dose may be administered if it has been at least 4 months after the first booster dose [25].

#### Prevention of AML Recurrence

Advances in AML have improved survival outcomes but have not fully prevented disease relapse [26]. The most frequent cause of relapse is the incomplete eradication of leukemia clones after treatment [27]. Measurable residual disease (MRD) detects leukemia cells below the morphology threshold and is a biomarker for prediction, prognosis, monitoring in AML, and potential surrogate endpoint [28–30]. The detection of any persistent mutation after treatment, while being in complete remission is associated with a 34.4–48.2% incidence of relapse [31]. A systematic review demonstrated an estimated overall survival of 68% without MRD versus 34% for those with MRD [32•].

The methodologies for MRD include multiparameter flow cytometry (MFC), real-time quantitative PCR (qPCR), and or next-generation sequencing (NGS) [30]. MRD is assessed at different timepoints following induction and consolidation therapy and after completion of treatment [33]. MFC is applicable to most AML patients and identifies surface markers that are present in leukemia cells. Limitations of MFC include lack of standardization, reliance on high quality marrow sample, and sensitivity and gaps in data analysis [34]. PCR methods are highly sensitive and applicable to about 60% of AML patients [34]. The largest limitation for PCR is its restriction to patients with specific gene aberrations and recurrent mutations [35]. NGS is a high-throughput sequencing technology that allows for rapid, precise, and cost-effective sequencing of multiple genes, aiding in classification, prognostic stratification, treatment, and response assessment of AML [36]. Some current limitations of NGS are limited sensitivity and specificity of the assays and the inability to accurately discern between residual leukemia and clonal hematopoiesis [37, 38]. Heuser et al. showed that an NGS-MRD for non-DTA mutations (DNMT3A, TET2, ASXL1) was prognostic in AML patients after allo-HSCT [38]. Novel technologies such as single-cell sequencing (SCS) overcome the limitations of MRD in differentiating malignant versus pre-leukemic clones, allowing the early identification of AML relapse [39], and increasing the MRD detection at 0.12%. Machine learning [40], immunophenotype, and proteogenomics added to SCS could improve MRD detection and the dynamics of clonal evolution in AML survivors.

#### Patient-Reported Outcomes and Health-Related Quality of Life

Patient-reported outcomes (PROs) are an important aspect of survivorship. AML survivors report chemotherapy and stem cell transplant side effects, along with functional status and quality of life [41]. PROs are more accurate in capturing symptoms than health care provider assessments [42]. Health-related quality of life (HRQOL) PROM evaluates on an individual scale of physical and mental health perceptions and correlates to a person’s health risk, comorbid conditions, functional status, social support, and socioeconomic status. HRQOL in AML survivorship has been studied using instruments such as EORTC QLQ-30, FACT Leukemia (FACT-Leu), EQ-VAS, EORTC QLQ-Leu, Hematological Malignancies-Patient-Reported Outcome (HM-PRO), and Acute Myeloid Leukemia-Quality of Life (AML-QOL), FACT-BMT, and SF-36 (Table 1). Compared to best supportive care, treatment with hypomethylating agents is associated with improvement in quality of life (QoL) [43, 44••]. Longitudinal assessments after treatment completion and in the post-allo-HCT setting are needed for survivorship evaluation. QoL improves after completion of initial chemotherapy and remains stable in post-remission treatment [45]. AML survivors have worse QoL compared to general population, and factors associated with it include financial burden, allo-HCT, and lack of social support. Messerer and Hsu demonstrated [46, 47] a negative impact in QoL after allo-HCT versus conventional treatment alone [48].

**Table 1:** Health-related quality of life and PRO in acute myeloid leukemia survivors

	Study population	Study measure	Findings
Peipert et al. [92]	Unfit for intensive chemotherapy; *n* = 317	FACT-LEU EQ-5D	FACT-Leu is a reliable and valid outcome measure for AML clinical trials for patients not eligible for intensive therapy
Buckley et al. [93]	Undergoing intensive chemotherapy; *n* = 50	AML-QOL	AML-QOL is a reliable and sensitive patient-reported instrument to capture information about the symptoms and QOL experienced by patients with AML
Dombret et al. [94]	Patients randomized to azacitidine vs. conventional care; *n* = 291	EORTC QLQ-C30	No HRQoL detriment was seen with azacitidine or CCR at the group level during treatment
Leunis et al. [48]	AML survivors; *n* = 92	EORTC QLQ-C30 and EQ-5D	HRQoL in AML survivors is worse than the HRQoL in the general population
Pratz et al. [95]	Unfit for intensive chemotherapy (venetoclax added to azacitidine or low dose ara-c; *n* = 642	EORTC QLQ-C30 GHS/QLS PROMIS EQ-5D-5L HS-VAS	Venetoclax had a positive impact on HRQoL in patients with AML ineligible for intensive chemotherapy, leading to longer preservation of overall health status
Montesinos et al. [96]	Ineligible for intensive induction chemotherapy (addition of ivosidenib to azacitidine); *n* = 135	EORTC QLQ C30 GHS/QoL	Better HRQoL with ivosidenib and azacitidine across all EORTC QLQ-C30 subscales

Abbreviations: *EORTC QLQ-C30* European Organization for Research and Treatment of Cancer quality of life questionnaire, *EQ-5D-5L* EuroQoL 5-Dimension 5-Level, *GHS* global health status, *PROMIS* Patient-Reported Outcomes Measurement Information System, *HRQoL* health-related quality of life, *VAS* visual analog scale, *FACT-Leu* Functional Assessment of Cancer Therapy-Leukemia

Since clinical trials are limited to a selective number of patients, real-world data studies in PROs are needed to demonstrate the true impact of AML interventions in the community [49].

### Psychosocial

#### Mental Health

AML survivors after intensive treatment or allo-HCT experience psychological and cognitive effects negatively affecting QoL and clinical outcomes [50]. Depression and anxiety were present in up to 22% and 11.1% of AML patients after being 2 or more years in remission [51]. Up to 28% of patients with AML experienced posttraumatic stress disorder (PTSD) [52]. In HCT survivors, the rates of PTSD are between 12 and 30% [53]. In a study of 236 adult years after HSCT, psychological distress was present in 43% of allo-HCT survivors [54]. Significant and major depression was often linked to lower levels of physical function and less satisfaction with social support prior to transplant, which impaired physical and emotional recovery after allo-HCT [55]. Fear of recurrence is a risk factor for mental health concerns in leukemia survivors [56]. Improvement of mental health concerns gradually occurs over 1 to 5 years but can persist for longer than 20 years post-HCT [55, 57].

Fewer comorbidities, higher family income, working or being in school, and having a solid support system in place are associated with a higher emotional well-being [58].

Cognitive dysfunction has been evaluated in a few studies in older adults with AML. The estimated prevalence on cognitive dysfunction in hematologic cancer survivors was 17.2% in working memory, 35.2% in executive function, and impairment in working memory was associated with worsening survival [59]. AML chemotherapy did not have impact in cognitive or functional status [60, 61].

The NCCN survivorship guidelines have implemented screenings for evaluation of mental health, as well as pharmacological and non-pharmacological interventions [21]. The approach is multidisciplinary and requires neuropsychological evaluation, psychotherapy, and medications. Buchbinder and Khera [59] consider a patient-centered approach involving multiple stakeholders to improve psychosocial concerns after HCT.

#### Financial Burden

The cost of treatment for adult AML patients is an estimated $1.5 billion and $500 million respectively [62]. The costs of AML chemotherapy followed by allo-HCT are approximately $352,682 [63]. This often leads to financial and employment stressors in long-term cancer survivors. Financial burden or toxicity is often defined as any difficulty paying medical bills, high financial distress, cost-related medication nonadherence, food insecurity, and foregone or delayed care because of cost [64]. Financial toxicity is often multifactorial arising from several factors including socioeconomic status and demographics, health insurance, cancer treatment (medical, surgical, radiation, and supportive care), and end of life care [65]. There are very few studies evaluating financial costs and burden in AML (Table 2).

**Table 2:** Financial costs in acute myeloid leukemia

Author	Sample	Study design	Instruments	Results
Knight et al. [97]	*N* = 106	Single-institution survey, AML, and ALL	PROMIS Global 10, COST measure	58% had FT Factors: age > 65, African American versus Caucasian, Medicaid versus commercial insurance. Correlation with PROMIS global physical/mental and FT score. Lower FT score associated with lower mental and physical scores
Han et al. [98]	N: 5.5 million > 60 yo	Longitudinal study Korean National Health Insurance Service-Senior (NHIS-Senior) in South Korea AML	Database	High-intensity chemo had longer hospital stay and higher treatment expenses compared to low0intensity chemotherapy but was not statistically significant
Pandya et al. [99]	*N* = 1542	Retrospective cohort analysis	IQVIA Real World Database	Costs were highest in relapsed/refractory AML ($439,104), followed by HSCT ($ 329,621), induction ($198,657), and consolidation ($53,081) CV complications were associated with higher costs
Meyers et al. [100]	*N* = 4058 > 65 years	Retrospective SEER database Medicare	SEER	43% received chemotherapy, 57% supportive care only. 69.1% (CT) vs 95% (SC) died within a year Age 65–74 and CCI = 2 or 3 associated with receipt of chemotherapy. Mean all cause costs were $ 96,078, 76.3% inpatient utilization
Preussler et al. [101]	*N* = 991 CT only = 774, allo-HCT = 85 50–64 years AML chemotherapy alone or allo-HCT	Retrospective MarketScan database US private insurance	MarketScan database inpatient and outpatient	Adjusted mean 1-year costs were $280,788 for chemotherapy and $544,178 for allo-HCT. Patients on CT alone had a mean of 4 hospitalizations, 52.9 inpatient days, and 52.4 outpatient visits in year 1; Allo-HCT had 5 hospitalizations, 92.5 inpatient days, and 74.5 outpatient visits. AML in the first year has substantial healthcare costs and utilization

Abbreviations: *PROMIS Global 10 FT* Patient-Reported Outcomes Measurement Information System Financial Toxicity SEER: *CT* chemotherapy, *allo-HCT* allogenic hematopoietic stem cell transplant, *CV* cardiovascular, *CCI* charlson comorbidity index

Patients who experience cancer-related financial problems often reported a poor quality of life that affects their physical health, mental health, and social life [66]. One multi-site study looking at financial hardship after HSCT, even in patients with health insurance, found that 41% of patients had difficulty paying for transportation, 36% had difficulty meeting costs associated with changes in their home environment, and 19% had difficulty paying to relocate closer to their transplant center [67].

Along with financial burden, employment can also be distressing for patients. Among 1-year AML survivors, the percentage of full-time employment improves over time with very few limitations in productivity [68•]. Approximately 50% of patients can return to work after allo-HCT [69]. HSCT survivors have concern about employment, such as keeping their job, worrying about taking a leave of absence, or even the need to go on disability [9]. Barriers to returning to work after allo-HCT include the physical and mental impact from their disease and treatment, having difficulties performing job tasks due to side effects and concern about work relationships [70]. Unemployed post-allo-HCT survivors reported fatigue, pain, poorer perception of health, and lower quality of life compared to employed peers [71]. There is a great need to further evaluate financial toxicity among AML and HSCT survivors and to utilize resources to make treatment more affordable.

#### Post-allogeneic Stem Cell Transplant Effects

Late complications and chronic health conditions can appear early after allo-HCT with clinical consequences on long-term outcomes. These complications are not unique to AML patients and can be consequences of pre- and posttransplant comorbidities, chemotherapy and conditioning regimens, psychosocial, chronic GVHD, second malignances, bone disease, and infectious complications leading to decreased survival [72, 73]. Studies in different age groups showed the initial negative impact in QoL in early post-allo-HCT setting and further improvement in the long-term QoL. Chronic GVHD causes significant impairments related to physical functioning, sexual dysfunction, and greater psychosocial dysfunction [74]. These late complications and risk for developing chronic health conditions should be adequately managed by a patient’s clinician at least every 6 months with appropriate labs and diagnostic testing [23].

### Survivorship Care Delivery

#### AML Survivorship Guidelines

The American Cancer Society, American Society of Clinical Oncology, and the National Comprehensive Cancer Network (NCCN) have survivorship guidelines not specific for AML. [75]. Although MD Anderson Cancer Center has an Acute Myeloid Leukemia Survivorship Algorithm [76], these guidelines can be daunting to both patients and clinicians as they are extremely lengthy and difficult to navigate; they may not be applicable to a particular clinical context and could be subject to conflict of interest [77].

The ideal guidelines should incorporate high level of evidence recommendations extensive to different clinical and socioeconomic scenarios, considering the patient’s perspective.

#### Survivorship Care Plans

OncoLife, developed by Livestrong and the University of Pennsylvania, constitutes a valuable tool for individualized care plans in AML. OncoLife provides customized information and recommendations for patients at any phase of their survivorship journey. Providers enter information regarding a patient’s disease state, chemotherapy and allo-HSCT, side effects, and complications, and an individualized care plan is created that can be printed out for both patient and provider [78]. The information in the created plan is broken down by related risks and side effects to treatments received, future screening recommendations, healthy living tips, and psychosocial issues that may arise. Although there are no current studies evaluating the impact of survivorship care plan (SCP) in AML not undergoing transplant, Majhail et al. [79] demonstrated the positive impact in improvement in distress and Mental Component Summary of QoL in 52 AML allo-HCT survivors.

#### Survivorship Clinics

Clinicians play a key role by guiding AML patients through treatment, disease monitoring, and long-term survivorship [80]. Very few NCI designated cancer centers offer a survivorship clinic especially targeting adult survivors [81]. Several models of survivorship care programs exist, ranging from centralized to decentralized approach to a mixed model [82]. Telemedicine is being incorporated to facilitate cross-regional care; however, unreliable technology, not having computer access, and low computer literacy can make this method challenging in some rural areas [83]. The Hematologic Disorders ECHO program is a tele-monitoring model of best practices in diagnosis, management, surveillance, and long-term follow-up of acute leukemias as well as other hematologic malignancies [84]. Project Echo Survivorship coordinates survivorship care and delivery of recommended services for community providers in Texas [85]. There is an ongoing observational study of telemedicine in elderly AML patients, caregivers, and oncologists [86].

#### The Role of the PCP in AML Survivorship

Effective AML survivorship requires the partnership between the cancer institution and the primary care physician (PCP). However, many PCPs are unfamiliar with Cancer Survivorship guidelines and long-term chemotherapy and transplant complications. A survey study demonstrated barriers in the interactions between the referral center and the PCP; PCPs rarely to never received a treatment summary, a third rarely to never received timely updates, and about a third had difficulty transferring patient care responsibilities [87]. Other limitations include lack of resources to facilitate care, lack of awareness in screening and/or prevention guidelines and psychosocial needs, and inadequate time [88].

In general, PCPs felt comfortable providing general medical care and general cancer screening for survivors, however, felt that the primary oncologist would be better suited to provide care regarding medical concerns related to the cancer treatment and screening for cancer reoccurrence or secondary malignancies [87]. As PCPs play a pivotal role in providing survivor follow-up care, a survivorship care plan should be well established and laid out for each patient which include their diagnosis, treatment summary, and plan for follow-up medical care.

#### The Role of Caregivers in AML Survivorship

Family caregivers play a pivotal role in providing supportive care to a cancer survivor, which aids the survivor in coping and adjustment; however, it often comes at a considerable cost to the caregivers such as psychiatric comorbidity, compromising their physiological functioning [89]. As cancer survivors are increasingly dependent on their caregivers for physical and emotional support, caregivers have been noted to experience anxiety, distress, and depression equivalent or worse to that of their partner with cancer [90]. Extensive caregiver responsibilities have also contributed to an increase rate of depression, worse self-care, and increased chronic illnesses [6]. Literature and information are lacking about the interrelationship between cancer survivors and caregivers’ quality of life. There is also minimal literature and support resources for caregivers in general caring for a cancer survivor.

#### Challenges and Future Directions

Fatigue, infertility, and sexual dysfunction are one of the most common symptoms in AML survivors. Biomarkers of fatigue and correlation with HRQoL instruments, as well as therapeutic interventions, are needed. Further studies need to be done to determine the impact of non-pharmacological interventions such as psychosocial intervention, exercise, and complementary medicine versus novel pharmacological approaches [12].

MRD constitutes a valuable prognostic tool in AML, but its role as surrogate endpoint is yet to be determined. Its use as therapy decision-making in real-world require the standardization of MRD methodologies and newer technologies for better prediction of AML recurrence.

AML clinical trials are incorporating PROs and HRQol as endpoints, timing for assessments and validation across different studies are needed. Table 3 summarizes the areas of focus and research priorities in AML survivorship. In the post-allo-HCT setting, long-term data collection and interventions to improve HRQoL in AML survivors are strongly needed.

**Table 3:** Recommended areas of focus for future research priorities in AML Survivorship

Area	Focus
Study design and measures	Real World data in AML survivorship after chemotherapy and post-HCT Validated patient-reported outcome measures as surrogate endpoints in AML studies Long-term data according to age groups and in underserved/minority population Ultrasensitive MRD tools for prediction of AML recurrence Targeted/immune therapies for prevention of AML relapse
Screening and assessment	Disease-oriented survivorship guidelines Individualized survivorship plans Patient-centered approach with participation of multiple stakeholders
Rehabilitation and intervention	Caregiver and community support options Federal policies for financial assistance to decrease AML costs and return to work Mental Health services covered by Health insurance
Education and awareness	Patient, PCP, and community awareness in late complications Educational activities for PCP and caregivers in survivorship

Establishing a network of survivorship services facilitate an individualized survivorship care plan with detailed information regarding a patient’s cancer history and treatment, define surveillance goals and schedules, and health priorities to ensure potential health problems do not get missed. Communication between oncologists and PCPs should remain decentralized and open, to allow for effective follow-up care for the AML survivors.

Clinical counseling and education should be greatly emphasized prior to starting any treatment. As anxiety and depression are linked to worse clinical outcomes, making sure AML patients undergo mental health screening, and get offered mental health and social support throughout treatment and into survival is crucial to improve PROs. The impact of the stress and depression in caregivers is not known.

The costs of AML treatment cause financial toxicity in survivors. Novel treatment options, along with hospitalizations and allo-HCT, will continue increasing costs. Shifting towards outpatient AML management, along with safer and effective options, will reduce financial burden and improve survival outcomes.

AML survivors pose unique challenges and needs. There is a strong need for disease-oriented survivorship guidelines with individualized SCPs. As new therapeutic options arise, survivorship aspects must be considered since initial treatment response, using PROM and HRQoL instruments at different timepoints.

In addition to academic and regional efforts to provide adequate resources for survivorships and caregivers, public health policies in cancer survivorship must direct efforts in implementing personalized information, assessment and referrals, and dissemination and implementing methods and interventions for survivors and caregivers [91].

## Conclusion

AML is an aggressive hematologic malignancy requiring intensive treatment measures that profoundly affects survivors HRQOL. As the number of AML survivors continues to grow, there is a need to improve survivorship care to improve survivors’ overall health, wellness, and QoL. PCPs and oncologists need to communicate adequately about a patient’s survivorship care plan, which should include surveillance testing for recurrent or subsequent primary cancers, disease prevention, management of comorbid conditions, and monitoring for physical and psychological effects of cancer treatment. Survivors and caregivers need to have resources available to them in how to navigate back into a new normalcy of life, including care for medical complications, coping mechanisms for mental health problems, resources for financial burden, and social reintegration.
